# Weight status and meeting the physical activity, sleep, and screen-time guidelines among Texas children: results from a population based, cross-sectional analysis

**DOI:** 10.1186/s12887-022-03488-8

**Published:** 2022-07-20

**Authors:** Geronimo Bejarano, Riley P. Brayton, Nalini Ranjit, Deanna M. Hoelscher, Danielle Brown, Gregory Knell

**Affiliations:** 1 Department of Epidemiology, Human Genetics, and Environmental Sciences, The University of Texas Health Science Center at Houston (UTHealth) School of Public Health, Houston, TX USA; 2 Michael & Susan Dell Center for Healthy Living, The University of Texas Health Science Center at Houston (UTHealth) School of Public Health, Austin, TX USA; 3 Center for Pediatric Population Health, Children’s Health & The University of Texas Health Science Center at Houston (UTHealth) School of Public Health, 2777 N Stemmons Fwy, Suite 8400, Dallas, TX 75207 USA; 4 Department of Health Promotion & Behavioral Sciences, The University of Texas Health Science Center at Houston (UTHealth) School of Public Health, Houston, TX USA; 5 Department of State Health Services, State of Texas, Austin, TX USA

**Keywords:** Pediatric obesity, Overweight, Sleep, Exercise, Screen time

## Abstract

**Background:**

Evidence suggests that the interactive effects of physical activity, screen-time and sleep are stronger than independent effects of these behaviors on pediatric obesity. However, this hypothesis has not been fully examined among samples of young school-aged children. The aim of this study is to determine the association of weight status with meeting the physical activity, screen-time, and sleep guidelines, independently and concurrently, among 2nd grade children.

**Methods:**

The Texas School Physical Activity and Nutrition Project collected parent-reported physical activity, screen-time, and sleep, and measured body height and weight on a statewide representative weighted sample (*n* = 320,005) of children. Weighted multivariable logistic regressions were used to assess associations of weight status (classified using age- and sex-specific body weight [kg]/height [m]^2^, based on International Obesity Task Force cutoffs) with meeting the physical activity, screen-time, and sleep guidelines, while controlling for relevant covariates (age, sex, race/ethnicity, comorbidities etc.).

**Results:**

A greater proportion of healthy weight children (9.9%) met the physical activity, screen-time, and sleep guidelines concurrently compared to children who are thin (3.3%), or children with overweight (5.7%), obese (3.5%), and morbid obesity (1.0%). Children who were thin (adjusted odds ratio [aOR]:0.40, 95% confidence interval [CI]: 0.10, 1.50), overweight (aOR = 0.75, CI: 0.33, 1.70), obese (aOR = 0.53, CI: 0.15, 1.81), and morbidly obese (aOR = 0.10, CI: 0.02, 0.28) had lower odds of concurrently meeting the guidelines compared to children with healthy weight.

**Conclusions:**

Among this representative sample of Texas children, weight status was associated with meeting physical activity, screen-time, and sleep guidelines. Future studies should aim to evaluate causal relations between these behaviors and weight status.

## Introduction

Short sleep duration, physical inactivity, and increased screen-time are independent risk factors for childhood obesity [1–5]. The combination of these deleterious behaviors influences childhood obesity status to a greater degree than what is observed of each behavior independently [6, 7]. In light of these findings, the American Academy of Pediatrics and World Health Organization established evidence-based guidelines which recommend that children accumulate at least 60 minutes of moderate intensity aerobic physical activity (PA) per 24 hours, limit their screen-time to less than 2 hours, and sleep for 9–11 hours (aged 6–13 years) [1, 8, 9]. However, recent studies have reported that a majority of children do not meet the guideline recommendations, and the prevalence of independently meeting the guidelines is decreasing globally [10–12].

Among children and adolescents in the United States (U.S.), 23, 33, and 86% independently meet the PA, screen-time, and sleep guidelines, respectively [13]. Only 12.9% of all U.S. children aged 6–11 years and 5% of U.S. high school adolescents aged 12–17 years meet all three of the guidelines concurrently [13, 14]. The lack of U.S. children concurrently meeting guidelines is of growing concern. Childhood obesity prevalence has increased nationally from 5.2% in 1974 to 19.3% in 2018 [15]. Of particular concern, the prevalence of childhood obesity in the state of Texas is among the highest in the U.S. In 2019 it was reported that 17.3% of Texas children have obesity [16]. Nearly 20 years ago, Perez and colleagues [17] found that Texas children who did not concurrently meet the PA guidelines (20–30 minutes of vigorous- to moderate- intensity activity on at least 3 or 5 days in the past week, respectively) and screen-time guidelines (2 hours or less per day) had higher odds of obesity (boys: odds ration [OR]: 1.40, 95% confidence interval [CI]: 1.01, 1.94; girls: OR: 1.26, 95% CI: 0.78, 2.04) than those who were normal weight. Other studies have also demonstrated independent associations between physical activity, sedentary behaviors, and sleep [4, 13, 14] with weight status in children, however, there remains a limited understanding of the health implications of concurrently engaging in recommended levels of these behaviors with various health outcomes. In particular, the level of understanding on this topic remains scant among population-based samples of young school-aged U.S. children.

Therefore, the aim of this study is to determine whether weight status is associated with meeting the PA, screen-time, and sleep guidelines, independently and concurrently, among a statewide representative sample of 2nd grade children in Texas. We hypothesize that children with unhealthy weight will be associated with lower odds of achieving the PA, screen-time, and sleep guidelines, independently and concurrently, compared to children with healthy weight. The results from this study will help in developing more knowledge and awareness on the association between 24-hour cycle behaviors and pediatric obesity [18].

## Methods

### Study design, data source and participants

This cross-sectional study was reported and all methods were carried out in accordance to the Strengthening the Report of Observational studies in Epidemiology (STROBE) guidelines [19]. This analysis was conducted using 2015–2016 survey data collected by the Texas School Physical Activity and Nutrition (SPAN) Project. A full description of the Texas SPAN study design and participants is available elsewhere [20]. Briefly, Texas SPAN is a cross-sectional, statewide representative, school-based cluster survey administered annually since 2000. Texas SPAN is designed to measure prevalence of obesity, nutrition, PA, sleep, screen-time, and other health behaviors among 2nd, 4th, 8th, and 11th grade children in Texas. The current study included 2nd grade children (*n* = 320,005) who participated in Texas SPAN during the 2015–2016 school year. Written child assent and informed consent were obtained from all Texas SPAN participants and primary caregivers to use their data for research purposes. This study was reviewed and approved by the Committee for the Protection of Human Subjects at the University of Texas Health Science Center at Houston (UTHealth) (HSC-SPH-00-056), the Texas Department of State Health Services Institutional Review Board (IRB# 04–062), and local school district review committees.

### Measures

The primary exposure of interest was body weight status. Standard protocols were used to measure and record height and weight among the participants. Body mass index (BMI) was calculated using body weight in kilograms divided by the square of height in meters. Weight status was further classified by age and sex healthy (BMI: 18.5–24.99), thin (BMI: < 18.5), overweight (BMI: 25.00–29.99), obesity (BMI: 30.00–39.99), and morbid obesity (BMI: > 35.00), according to International Obesity Task Force (IOTF) definitions [21].

The primary outcome variables of interest were meeting the PA, screen-time, and sleep guidelines, independently and concurrently. The participating child’s primary caregiver was asked to report on their child. PA behavior was assessed by reporting the total number of days in the last week the child performed activity that increased his/her heart rate and made him/her breathe hard some of the time for a total of at least 60 minutes. Those participants reporting 7-days of PA as described were considered to have met the PA guideline [8]. Screen-time behaviors were assessed by asking how many hours per day the child usually spends watching TV, DVDs, movies or using a computer, tablet/iPad® or Smartphone® away from school. The hours for each screen-based activity were summed. Children having less than 2 hours total of screen-time per day were considered to have met the screen-time guideline. Sleep was assessed by asking parents how many hours of sleep their child usually gets per night. Those children reporting 9–11 hours of sleep per night were considered to have met the sleep guideline [1, 8, 22]. The PA, screen-time, and sleep questions were adapted (for parent proxy report) from the Youth Risk Behavior Surveillance System (YRBSS) questionnaires and have been shown to demonstrate acceptable reliability [23].

Potential covariates that are known to be associated with weight status included in the analysis were caregiver-reported demographic characteristics (e.g. age [years]; sex; race/ethnicity [non-Hispanic White, non-Hispanic Black, Hispanic/Latinx, and other]; financial assistance (yes/no); healthy food preference (always, sometimes, or never); comorbidities [yes/no] including physical limitations, asthma, diabetes, attention deficit hyperactivity disorder, autism spectrum disorder), and parent BMI [continuous] [24–28].

### Statistical analysis

The proportions and 95% confidence intervals (CI) of weight status were estimated in sex strata by potential covariates (e.g., age, sex, race/ethnicity, financial assistance, healthy food preference, comorbidities, and parent BMI). Multiple logistic regressions in sex stratum were adjusted for potential covariates and used to calculate weighted relative odds of independently and concurrently meeting the PA, screen-time, and sleep guidelines based on child weight status. All statistical analyses were conducted in STATA 15.1 (Stata Statistical Software: Release 15, StataCorp LLC, College Station, TX).

## Results

Sample demographic and health characteristics are presented in Table 1. Among Texas 2nd grade children, nearly two-thirds (58.5%) had a healthy weight status, 4.7% were thin, 17.38% were overweight, 9.3% had obesity, and 9.6% had morbid obesity. The majority were 7 years of age (62.3%), Hispanic/Latinx (56.4%) ethnicity, and received financial assistance (70.3%). Asthma was the most common comorbidity (14.4%) followed by attention deficit hyperactivity disorder (7.8%), physical limitations (3.2%), autism spectrum disorder (0.9%), and diabetes (0.4%).

**Table 1:** Weighted estimates of demographic characteristics of students by sex among Texas 2nd grade children, School Physical Activity and Nutrition Survey, Texas, 2015–2016

Characteristic	% (95% CI)		
	Overall (***N*** = 320,005)	Girls (***n*** = 164,803)	Boys (***n*** = 155,502)
**Weight Status**^**a**^			
Healthy	58.5 (53.0, 63.8)	56.8 (51.0, 62.5)	60.1 (52.4, 67.2)
Thin	4.7 (3.2, 6.7)	4.9 (3.0, 8.1)	4.5 (2.6, 7.7)
Overweight	17.8 (15.0, 21.0)	18.1 (14.0, 23.0)	17.5 (13.8, 22.0)
Obese	9.3 (7.4, 11.7)	9.4 (7.1, 12.3)	9.3 (6.5, 13.1)
Morbidly obese	9.6 (7.3, 12.7)	10.8 (7.8, 15.0)	8.6 (6.0, 12.2)
**Age, years**			
7	62.3 (55.5, 68.6)	63.3 (55.2, 70.7)	61.4 (55.0, 67.5)
8	35.4 (29.5 41.8)	34.5 (27.7, 42.1)	36.2 (30.4, 42.5)
9	2.3 (1.4, 3.7)	2.2 (1.1, 4.4)	2.4 (1.5, 3.8)
**Race/ethnicity**			
NH White	23.5 (14.8, 35.4)	23.9 (15.0, 35.8)	23.2 (14.2, 35.5)
NH Black	10.8 (5.6, 19.8)	10.3 (5.5, 18.4)	11.3 (5.5, 21.6)
Hispanic/Latinx	56.4 (44.0, 68.1)	56.9 (46.1, 67.1)	55.9 (41.5, 69.4)
Other^b^	9.3 (6.5, 13.0)	8.9 (5.8, 13.4)	9.6 (5.3, 16.7)
**Financial assistance**			
No	29.7 (21.1, 40.0)	29.9 (21.4, 40.1)	29.4 (19.9, 41.1)
Yes	70.3 (60.0, 78.9)	70.1 (59.9, 78.6)	70.6 (58.9, 80.1)
**Healthy food preference**			
Always	6.9 (5.2, 9.1)	5.5 (3.9, 7.7)	8.2 (5.8, 11.4)
Sometimes	48.5 (44.8, 52.3)	47.4 (41.7, 53.2)	49.6 (45.2, 54.0)
Never	44.6 (40.5, 48.7)	47.1 (41.1, 53.1)	42.2 (38.4, 46.2)
**Comorbidities**^**c**^			
Physical limitations	3.2 (2.1, 4.8)	2.4 (1.4, 3.9)	4.0 (2.2, 7.0)
Asthma	14.4 (11.8, 17.4)	10.9 (8.1, 14.5)	17.6 (13.9, 22.1)
Diabetes	0.4 (0.2, 0.9)	0.6 (0.2, 1.8)	0.3 (0.1, 0.8)
ADHD	7.8 (6.2, 9.8)	3.8 (2.6, 5.4)	11.6 (9.0, 14.7)
ASD	0.9 (0.6, 1.5)	0.6 (0.2, 1.5)	1.3 (0.7, 2.2)
**Parent BMI (mean, SD)**	28.75 (27.87, 29.63)	28.93 (28.32, 29.54)	28.60 (27.17, 30.02)

*Abbreviation*: *CI* Confidence interval, *NH* Non-Hispanic, *ADHD* Attention deficit hyperactivity disorder, *ASD* Autism spectrum disorder, *BMI* Body mass index, *SD* Standard deviation

^a^Defined using IOTF age-specific cutoffs [healthy (BMI: 18.5–24.99), thin (BMI: < 18.5), overweight (BMI: 25.00–29.99), obese (BMI: 30.00–39.99), and morbid obese (BMI: > 35.00)]

^b^Includes Asian, Native Hawaiian/Pacific Islander and non-Hispanic multiple race/ethnicities; ^c^ Proportion reported is for those where the comorbidity is present

The prevalence of children who independently met the PA, screen-time, and sleep guidelines differed by weight status and gender (Table 2). Specifically, 4.9% (95% CI: 2.3, 10.2) of girls with morbid obesity met the PA guideline, compared to 24.5% (95% CI: 19.7, 29.9) of girls with healthy weight. Among boys, 15.8% (95% CI: 8.6, 27.2) of boys with obesity met the PA guideline, compared to 27.5% (95% CI: 23.0, 32.6) of boys with healthy weight. For screen-time and sleep, results followed a similar pattern overall and by sex. The prevalence of meeting the guidelines concurrently was lowest among those children classified as having obesity and morbid obesity (overall and by sex), with the exception of boys with thin weight status (2.2, 95% CI: 0.3, 18.2). Overall, 3.5% (95% CI: 1.2, 10.0) and 1.0% (95% CI: 0.3, 2.9) of children with obesity and morbid obesity met the guidelines concurrently, compared to 9.9% (95% CI: 6.9, 14.1) of children with healthy weight. Among girls with obesity and morbid obesity, 3.5% (95% CI: 1.1, 11.3) and 1.1% (95% CI: 0.3, 4.1) concurrently met the guidelines, compared to 8.3% (95% CI: 5.5, 12.3) of girls with healthy weight. Meanwhile, 3.6% (95% CI: 0.6, 18.2) of boys with obesity and 0.08% (95% CI: 0.1, 5.9) of boys with morbid obesity concurrently met the guidelines, compared to 11.3% (95% CI; 6.8, 18.2) of boys with healthy weight.

**Table 2:** Weighted prevalence of meeting physical activity, sleep, and screen-time recommendations independently and in combination by weight status among Texas 2nd grade children, School Physical Activity and Nutrition Survey, 2015–2016

Behavior	Meeting guidelines^**a**^, % (95% CI)				
	Healthy (***n*** = 176,643)	Thin (***n*** = 15,360)	Overweight (***n*** = 58,561)	Obese (***n*** = 33,920)	Morbid Obese n (35,521)
**Overall**					
*Independent*					
PA	26.1 (22.7, 29.8)	20.1 (10.1, 35.8)	19.3 (14.2, 25.7)	10.9 (6.8, 17.1)	10.0 (6.2, 15.5)
Sleep	71.6 (63.6, 78.5)	63.6 (48.5, 76.5)	65.3 (54.4, 74.9)	65.3 (51.9, 76.7)	59.2 (48.4, 69.1)
ST	38.6 (30.2, 47.7)	35.3 (21.1, 52.5)	41.7 (31.6, 52.5)	29.7 (19.8, 42.0)	28.2 (18.5, 40.5)
*Combinations*					
PA + sleep	19.8 (16.6, 23.6)	12.6 (6.2, 23.7)	13.1 (8.8, 19.0)	7.6 (3.9, 14.3)	4.6 (2.3, 8.7)
PA + ST	12.8 (9.2, 17.3)	4.5 (1.5, 12.2)	7.4 (4.3, 12.6)	5.3 (2.4, 11.2)	2.2 (0.9, 6.0)
Sleep + ST	32.0 (23.7, 41.7)	30.4 (16.8, 48.6)	32.1 (21.4, 45.1)	21.7 (12.2, 35.6)	17.9 (9.5, 31.2)
PA + sleep + ST	9.9 (6.9, 14.1)	3.3 (1.0, 10.9)	5.7 (2.7, 11.8)	3.5 (1.2, 10.0)	1.0 (0.3, 2.9)
**Girls**					
*Independent*					
PA	24.5 (19.7, 29.9)	17.0 (6.4, 38.1)	13.8 (8.3, 22.1)	9.5 (5.1, 17.2)	4.9 (2.3, 10.2)
Sleep	71.0 (63.3, 77.4)	56.7 (35.2, 75.9)	66.6 (52.4, 78.3)	57.4 (44.8, 69.0)	57.3 (39.3, 73.6)
ST	38.1 (29.8, 47.1)	30.6 (15.7, 50.9)	51.5 (35.8, 67.1)	37.6 (25.6, 51.3)	45.3 (30.2, 61.4)
*Combinations*					
PA + sleep	19.3 (14.5, 25.2)	8.6 (3.4, 19.9)	9.0 (4.5, 17.1)	5.3 (2.2, 12.3)	1.8 (0.7, 4.5)
PA + ST	10.7 (7.5, 15.1)	4.3 (0.9, 17.8)	6.7 (2.6, 16.2)	6.4 (2.7, 14.5)	3.4 (1.2, 9.4)
Sleep + ST	30.6 (22.8, 39.7)	22.6 (9.6, 44.5)	40.7 (22.8, 61.4)	26.2 (14.8, 42.0)	31.2 (16.7, 50.5)
PA + sleep + ST	8.3 (5.5, 12.3)	4.3 (0.9, 17.8)	5.6 (1.8, 16.2)	3.5 (1.1, 11.3)	1.1 (0.3, 4.1)
**Boys**					
*Independent*					
PA	27.5 (23.0, 32.6)	23.1 (11.1, 42.0)	24.6 (16.3, 35.5)	12.3 (5.9, 23.7)	15.8 (8.6, 27.2)
Sleep	72.3 (62.5, 80.3)	70.3 (47.6, 86.0)	64.2 (53.4, 73.7)	73.1 (52.0, 87.2)	61.3 (42.6, 77.2)
ST	39.0 (29.0, 50.1)	40.2 (20.2, 64.0)	31.7 (21.1, 44.5)	22.7 (11.1, 40.8)	7.8 (3.4, 16.6)
*Combinations*					
PA + sleep	20.3 (16.2, 25.2)	16.3 (5.6, 39.2)	17.0 (10.0, 27.3)	9.9 (4.0, 22.3)	7.9 (3.7, 16.1)
PA + ST	14.5 (9.5, 21.6)	4.6 (1.0, 18.1)	8.2 (3.3, 18.9)	4.4 (1.0, 16.8)	0.8 (0.1, 5.8)
Sleep + ST	33.2 (23.1, 45.1)	38.7 (18.4, 63.7)	23.5 (14.1, 36.4)	17.5 (7.3, 36.4)	2.4 (0.8, 6.7)
PA + sleep + ST	11.3 (6.8, 18.2)	2.2 (0.3, 15.3)	5.8 (1.6, 18.6)	3.6 (0.6, 18.2)	0.8 (0.1, 5.9)

*Abbreviation*: *CI* Confidence interval, *PA* Physical activity, *ST* Screen time

^a^Defined using IOTF age-specific cutoffs [healthy (BMI: 18.5–24.99), thin (BMI: < 18.5), overweight (BMI: 25.00–29.99), obese (BMI: 30.00–39.99), and morbid obese (BMI: > 35.00)]

The adjusted odds ratio (aOR) of meeting the PA, screen-time, and sleep guidelines among Texas 2nd grade children, after controlling for age, race/ethnicity, financial assistance, comorbidities, and parent BMI, are presented overall and by sex in Table 3. There were significant associations with having obesity and morbid obesity and independently meeting the PA guideline. Specifically, girls with morbid obesity had 0.16 (95% CI: 0.05, 0.47) and girls with obesity had 0.37 (95% CI: 0.18, 0.77) times the odds of meeting the PA guideline compared to girls with healthy weight. For boys, those classified as having morbid obesity and obesity had 0.42 (95% CI: 0.18, 0.97) and 0.41 (95% CI: 0.18, 0.92) times the odds of meeting the PA guidelines, respectively, compared to boys with healthy weight.

**Table 3:** Adjusted relative odds of meeting physical activity, sleep and screen-time recommendations independently and in combination by weight status among Texas 2nd grade children, School Physical Activity and Nutrition survey, 2015–2016

Behavior	Odds Ratio^**a**^ (95% CI) of Unhealthy Weight Status^**b**^				
	Healthy	Thin	Overweight	Obese	Morbid Obese
**Overall**					
*Independent*					
PA	Ref.	0.75 (0.29, 1.92)	0.76 (0.50, 1.16)	0.39 (0.23, 0.69)	0.28 (0.14, 0.59)
Sleep	Ref.	0.76 (0.38, 1.51)	0.72 (0.44, 1.16)	0.94 (0.49, 1.80)	0.87 (0.52, 1.45)
ST	Ref.	1.10 (0.43, 2.81)	1.15 (0.71, 1.85)	0.74 (0.35, 1.55)	0.76 (0.44, 1.31)
*Combinations*					
PA + sleep	Ref.	0.57 (0.23, 1.40)	0.67 (0.37, 1.19)	0.43 (0.22, 0.87)	0.24 (0.10, 0.58)
PA + ST	Ref.	0.39 (0.11, 1.31)	0.70 (0.33, 1.47)	0.53 (0.20, 1.39)	0.24 (0.07, 0.80)
Sleep + ST	Ref.	1.26 (0.44, 3.63)	0.97 (0.60, 1.57)	0.73 (0.29, 1.83)	0.81 (0.40, 1.63)
PA + sleep + ST	Ref.	0.40 (0.10, 1.50)	0.75 (0.33, 1.70)	0.53 (0.15, 1.81)	0.10 (0.02, 0.48)
**Girls**					
*Independent*					
PA	Ref.	0.84 (0.26, 2.72)	0.55 (0.28, 1.06)	0.37 (0.18, 0.77)	0.16 (0.05, 0.47)
Sleep	Ref.	0.63 (0.27, 1.48)	0.75 (0.40, 1.40)	0.75 (0.42, 1.36)	1.11 (0.49, 2.52)
ST	Ref.	0.95 (0.37, 2.41)	1.46 (0.69, 3.09)	1.04 (0.55, 1.99)	1.98 (1.06, 3.69)
*Combinations*					
PA + sleep	Ref.	0.51 (0.18, 1.44)	0.45 (0.20, 1.00)	0.29 (0.10, 0.82)	0.07 (0.02, 0.22)
PA + ST	Ref.	0.49 (0.85, 2.80)	0.59 (0.23, 1.50)	0.87 (0.29, 2.60)	0.52 (0.13, 2.05)
Sleep + ST	Ref.	0.85 (0.28, 2.58)	1.08 (0.47, 2.45)	0.99 (0.87, 4.58)	1.99 (0.87, 4.58)
PA + sleep + ST	Ref.	0.65 (0.12, 3.53)	0.62 (0.21, 1.81)	0.67 (0.15, 3.02)	0.08 (0.19, 0.33)
**Boys**					
*Independent*					
PA	Ref.	0.71 (0.25, 2.01)	0.98 (0.51, 1.87)	0.41 (0.18, 0.92)	0.42 (0.18, 0.97)
Sleep	Ref.	0.87 (0.31, 2.45)	0.64 (0.36, 1.13)	1.19 (0.42, 3.39)	0.71 (0.30, 1.70)
ST	Ref.	1.23 (0.33, 4.50)	0.81 (0.47, 1.41)	0.52 (0.17, 1.54)	0.11 (0.04, 0.30)
*Combinations*					
PA + sleep	Ref.	0.64 (0.17, 2.49)	0.98 (0.50, 1.93)	0.57 (0.23, 1.42)	0.48 (0.19, 1.21)
PA + ST	Ref.	0.33 (0.06, 1.84)	0.70 (0.27, 1.85)	0.35 (0.07, 1.79)	0.07 (0.01, 0.61)
Sleep + ST	Ref.	1.67 (0.40, 7.03)	0.75 (0.35, 1.63)	0.53 (0.15, 1.86)	0.09 (0.03, 0.27)
PA + sleep + ST	Ref.	0.22 (0.26, 1.94)	0.83 (0.31, 2.19)	0.47 (0.07, 3.19)	0.12 (0.14, 1.09)

*Abbreviation*: *CI* Confidence interval, *PA* Physical activity, *ST* Screen time

^a^Adjusted for age, race/ethnicity, financial assistance, healthy food preference, parent overweight/obesity status

^b^Defined using IOTF cutoffs [healthy (BMI: 18.5–24.99), thin (BMI: < 18.5), overweight (BMI: 25.00–29.99), obese (BMI: 30.00–39.99), and morbid obese (BMI: > 35.00)]

Children with obesity and morbid obesity of both sexes were less likely to concurrently meet the guidelines than children with healthy weight. Specifically, girls with morbid obesity had lower odds (aOR: 0.52, 95% CI: 0.13, 2.05) of achieving the PA and screen-time guidelines concurrently compared to girls with healthy weight. Further, girls with morbid obesity had 0.08 (95% CI: 0.19, 0.33) times the odds of achieving all three of the guidelines concurrently compared to girls with healthy weight. Boys with morbid obesity had 0.09 (95% CI: 0.03, 0.27) times the odds of meeting the sleep and screen-time guidelines concurrently.

## Discussion

This study found that weight status and sex are associated meeting the PA, screen-time, and sleep guidelines, independently and concurrently. The prevalence of meeting the PA guideline was over three times higher among boys with morbid obesity compared to girls with morbid obesity. There are several potential explanations for the observed sex difference. In a prospective study of children aged 8 to 12 years, boys were found to be 19% more physically active than girls and these differences were attributed to less extracurricular sport participation, less parental support, and less perceived competence in physical education in girls compared to boys [29]. In other studies of younger children (aged 4 to 6 years and 9 to 11 years, respectively), boys have been found to be more physically active compared to girls, regardless of obesity status [30, 31]. Conversely, nearly half of girls with morbid obesity met the screen-time guidelines compared to less than 10% of boys with morbid obesity. Thirty one percent of girls with morbid obesity and 2.4% of boys with morbid obesity met the screen-time and sleep guidelines. Despite these differences, a similar proportion of boys and girls met all three guidelines concurrently, suggesting that while prevalence of concurrently meeting guidelines is of concern among all children, different problematic movement behavior patterns may be at play among male versus female children. Overall, children with healthy weight had the highest prevalence of meeting any of the guidelines, independently and concurrently, compared to children with unhealthy weight. Similar trends were observed when evaluating the odds of meeting any of the guidelines by weight status. For instance, none of the boys with unhealthy weight were more likely to meet any combination of the guidelines than boys with healthy weight with the exception of thin boys more likely to meet sleep and screen-time guidelines concurrently. Similarly, none of the girls with unhealthy weight were more likely to meet certain guidelines, independently and concurrently, compared to girls with healthy weight with the exception of thin girls and girls with morbid obesity more likely to meet sleep and screen-time guidelines concurrently. Most notably, girls with morbid obesity were nearly 2 times more likely to meet the screen-time guidelines independently, and the sleep and screen-time guidelines concurrently, compared to girls with healthy weight. This demonstrates the importance of studying and evaluating adherence to the guidelines concurrently by sex. Further, these results contribute to a limited body of research that suggests that meeting the PA, screen-time, and sleep guidelines concurrently may have an important relationship with pediatric obesity [6, 17, 18].

Overall, these results align with previous findings from population-based samples of U.S. adolescents, which reported that a very small proportion (5%) of students met all of the guidelines concurrently, [14] and those who do not meet the guidelines were more likely to have obesity.^17^ However, participants in our analysis were younger (2nd grade) than those included in similar studies [14, 17], which is important given the challenges and deleterious health outcomes related to early-onset morbid obesity in childhood. Similar to the current study’s findings, Laurson et al. [7] concluded that the relationship between weight status and meeting all of the guidelines appeared to be associated in a graded manner where those with the unhealthiest weight status had the lowest odds of meeting all of the guidelines. Overall, the literature suggests that adherence to the PA, screen-time, and sleep guidelines among children differs across sexes, and that these behaviors are associated with weight status.

The prevalence of children independently meeting the guidelines provided interesting findings. In particular, it appears that the low prevalence of meeting all of the guidelines concurrently was primarily driven by the inclusion of meeting the PA guidelines, rather than meeting the sleep or screen-time guidelines. For example, among children with healthy weight (boys and girls), 32.0% met the sleep and screen-time guidelines concurrently. When adherence to the PA guideline was added to the combination, the proportion of children meeting all of the guidelines dropped to 9.9%. This was consistently observed across all weight status categories and for both sexes when evaluated per strata. This may help explain the mechanisms by which children with obesity and morbid obesity are less likely to meet all three guidelines concurrently. Social norms (e.g., lower levels of adult support and self-conscious of body/looks when physically active) and psychological barriers (e.g., lack of interest, fear of being teased/chosen last, lack of energy/willpower/motivation) may disproportionately increase the barriers to meeting the physical activity guidelines for children with obesity and morbid obesity compared to children with healthy weight [32–34].

This study is not without limitations. First, the measures for the primary outcomes of interest, adherence to the PA, screen-time, and sleep guidelines, were reported by the child’s primary caregiver, which has mixed evidence of agreement compared to self-report in children [35, 36]. However, children in our study were very young (2nd grade) and the child’s primary caregiver report might be preferable in this age group. Future studies could aim to use objective PA and sleep measurement tools such as accelerometers, however the guidelines were developed using self-report measures which could indicate that measuring adherence to the guidelines with objective measurement tools is incorrect [37]. Although there is evidence of associations between screen-time, pediatric obesity [38], and other health outcomes [39] some of the guidelines (e.g., American Academy of Pediatrics) have removed previous screen-time recommendations due to potential weaknesses of screen-time measures, which include lack of evidence for a clear threshold of time and determining if screen-time is a proxy measure for sedentary behavior [40–42]. Even though screen-time measures have potential weaknesses, systematic reviews continue to report moderately strong evidence of association between screen-time and obesity and suggest more evidence is needed to inform guidelines [40]. Finally, given the cross-sectional design of this study, we are unable to make conclusions regarding the temporal associations between pediatric weight status and meeting the guidelines. Despite these limitations, a strength of this study is the large sample size which is state-representative and includes more low-income and diverse populations than the representative U.S. population. Additionally, our study used direct measurement of height and weight to calculate child BMI, which has been found to be more accurate than self-reported BMI [43].

## Conclusion

Finally, this study added to our understanding of how pediatric body weight status is associated with a 24-hour cycle of behaviors. Importantly, most studies to this point on this young of a population have only focused on a single behavior in isolation, thereby ignoring the inherent displacing effect of the behaviors on each other, and potential interactive effects of multiple behaviors occurring simultaneously. To our knowledge, this is the first study to report the association between concurrently meeting the PA, screen-time, and sleep guidelines with weight status in a population-based sample of young school-aged children. Although the results of this analysis indicate that PA in particular is strongly related to body weight status in this age group, future studies should strive to consider PA, sleep and sedentary behaviors both independently and concurrently in studies of their effects on pediatric obesity and other related health outcomes.

The results of this study should raise awareness to the potential magnitude of not adhering recommendations for 24-hour cycle of physical behaviors in pediatric populations and should inform future research to consider the interrelation of adherence to the PA, screen-time, and sleep guidelines when evaluating pediatric obesity. We found that adherence to the PA guidelines is much lower than adherence to the screen-time or sleep guidelines, therefore intervening on the PA guideline adherence may provide the most benefit. The difference in likelihood between concurrently meeting the guidelines and weight statuses suggests increased attention should be given to adherence to the guidelines in children with unhealthy weight compared to children with healthy weight.

## Data Availability

The datasets used and/or analysed during the current study are not publicly available due to concerns about analysis quality and consistency but are available from the corresponding author on reasonable request.

## Abbreviations

PA: Physical activity
U.S.: United States
SPAN: School Physical Activity and Nutrition
BMI: Body mass index
IOTF: International Obesity Task Force
CI: Confidence intervals
aOR: Adjusted odds ratio
STROBE: Strengthening the Report of Observational studies in Epidemiology
